# Progress in biomechanical stimuli on the cell-encapsulated hydrogels for cartilage tissue regeneration

**DOI:** 10.1186/s40824-023-00358-x

**Published:** 2023-03-20

**Authors:** Shiva Taheri, Hanieh Sadat Ghazali, Zahra Sadat Ghazali, Amitava Bhattacharyya, Insup Noh

**Affiliations:** 1 Convergence Institute of Biomedical Engineering and Biomaterials, Seoul National University of Science and Technology, Seoul, 01811 Republic of Korea; 2 Department of Nanotechnology, School of Advanced Technologies, Iran University of Science and Technology, Tehran, 1684613114 Iran; 3 Department of Biomedical Engineering, Amirkabir University of Technology, Tehran, 158754413 Iran; 4 Functional, Innovative, and Smart Textiles, PSG Institute of Advanced Studies, Coimbatore, 641004 India; 5 Department of Chemical and Biomolecular Engineering, Seoul National University of Science and Technology, Seoul, 01811 Republic of Korea

**Keywords:** Cartilage, Bioreactor, Hydrogel, Biomechanical stimuli, Regeneration

## Abstract

**Background:**

Worldwide, many people suffer from knee injuries and articular cartilage damage every year, which causes pain and reduces productivity, life quality, and daily routines. Medication is currently primarily used to relieve symptoms and not to ameliorate cartilage degeneration. As the natural healing capacity of cartilage damage is limited due to a lack of vascularization, common surgical methods are used to repair cartilage tissue, but they cannot prevent massive damage followed by injury.

**Main body:**

Functional tissue engineering has recently attracted attention for the repair of cartilage damage using a combination of cells, scaffolds (constructs), biochemical factors, and biomechanical stimuli. As cyclic biomechanical loading is the key factor in maintaining the chondrocyte phenotype, many studies have evaluated the effect of biomechanical stimulation on chondrogenesis. The characteristics of hydrogels, such as their mechanical properties, water content, and cell encapsulation, make them ideal for tissue-engineered scaffolds. Induced cell signaling (biochemical and biomechanical factors) and encapsulation of cells in hydrogels as a construct are discussed for biomechanical stimulation-based tissue regeneration, and several notable studies on the effect of biomechanical stimulation on encapsulated cells within hydrogels are discussed for cartilage regeneration.

**Conclusion:**

Induction of biochemical and biomechanical signaling on the encapsulated cells in hydrogels are important factors for biomechanical stimulation-based cartilage regeneration.

## Introduction

Human tissue has a limited capacity for self-regeneration, which has prompted widespread interest in tissue regeneration and sped up the development of regenerative medicine that can replace or fix damaged organs or tissues. To create functional replacements for injured tissues, tissue engineering combines the principles of biology and engineering. One of the main research directions in tissue engineering is the regulation of cell fate [1]. Multiple environmental signals, including biochemical stimuli, mechanical forces, biomaterial characteristics, scaffold properties and extracellular matrix properties, work together to promote tissue formation [2]. The requirements to create tissue constructions that can mimic native tissues in terms of function, architecture, composition, and dynamics are still present in studies on the regeneration of diseased and injured tissues [3].

There are ample evidences that cells are under specific conditions and exposed to different types of stress, including tensile, compressive, and shear stress in normal biological systems [4]. As examples, endothelial cells are more responsive to shear loads, fibroblasts are more responsive to tensile loads, and chondrocytes more responsive to compressive loads [5]. Some tissues, such as bone, cartilage, tendon, and dental tissues, are considered to be the most load-bearing tissues, which mean that the cells of those tissues encounter different loads and forces under physiological conditions [6, 7]. It can be concluded that for growth and remodeling or for maintaining biomechanical hemostasis of the load-bearing tissue, biomechanical factors should be considered in tissue engineering constructs [4, 5]. Moreover, traditional 2D culture has several limitations in mimicking the responses of patient tissues and organs, and 2D culture systems are unable to simulate specific cell characteristics [8]. In light of this, recent research has focused on 3D models that more closely resemble natural microenvironments [8, 9].

Cartilage is a connective tissue found in different parts of the human body. Based on the ECM component, it is categorized as hyaline, fibrous, and elastic cartilage [10, 11]. Elastic cartilage contains a high amount of elastin, which gives it biomechanical springiness and flexibility. It contains both collagen types I and II, and can be found in locations such as the outer ear and epiglottis [11]. Fibrocartilage is a strong and flexible connective tissue which is found in intervertebral discs and knee meniscus, and its main component is collagen type I. While fibrocartilage has low compressive and high tensile characteristics, hyaline cartilage has higher compressive strength and lower tensile strength [12]. Hyaline cartilage is the third and most common form of cartilage and mainly contains type II collagen. Further, it is divided into articular and non-articular hyaline cartilage. Articular cartilage can be found on the surface of articulating bones, but non-articular cartilage can be found on the nose, larynx, and end of the ribs [11]. Because collagen type I is a marker of fibrocartilage and hyaline cartilage contains little to no collagen type I, its presence of collagen type I poses a significant obstacle to the regeneration of articular cartilage [12, 13]. It is necessary to assess the amount of collagen type I/collagen type II in all cartilage regeneration studies, as a high collagen type I/collagen type II ratio indicates fibrocartilage formation rather than hyaline cartilage formation [12].

**Fig. 1:**
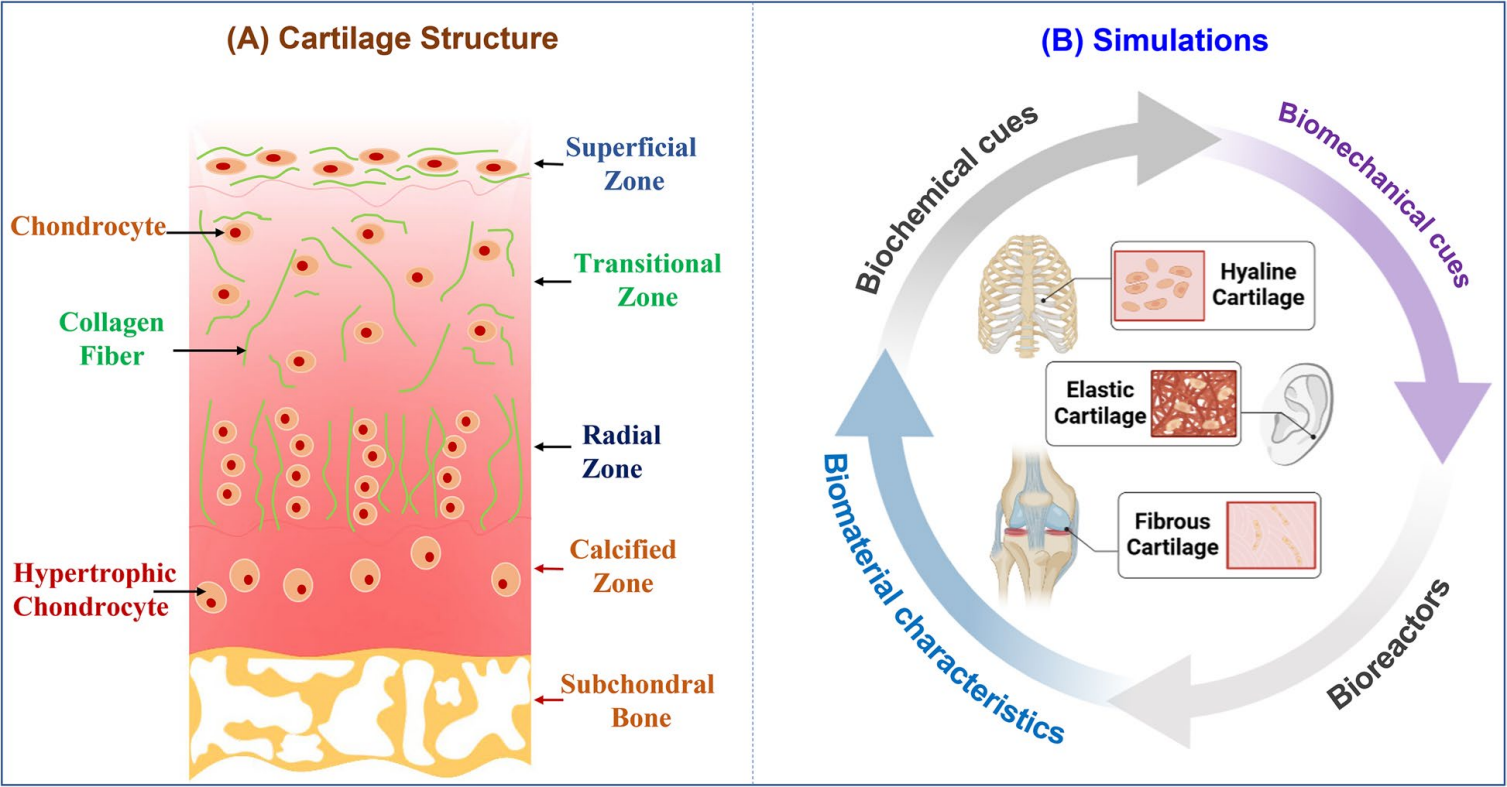
**A** Various zones and structure of articular cartilage, **B** Cartilage Tissue regeneration; different types of cartilage tissue and affective factors in tissue regeneration

The articular cartilage has substantial clinical significance because its damage can result in serious musculoskeletal dysfunction [14]. Cartilage has received considerable interest in tissue engineering because of its limited ability to heal and repair due to lack of blood supply. Articular cartilage is a load-bearing and avascular tissue with different zones that covers the bone surface as a smooth and lubricating layer to reduce wear during movement and distribute force along the joint (Fig. 1A) [10, 15]. Articular chondrocytes are the only cells in the healthy cartilage that produce and maintain the cartilaginous matrix. The matrix components provide articular cartilage with biomechanical properties [4, 16]. The extracellular matrix (ECM) and pericellular matrix (PCM) are the two principal components of articular cartilage. PCM aids in shielding cartilage chondrocytes from mechanical pressures, which is weak in osteoarthritis patients [17]. It is necessary to apply biomechanical stimulation of sufficient magnitude to develop and maintain healthy articular cartilage phenotypes [18]. Following cartilage under loading, chondrocytes undergo a variety of physiological changes, including hydrostatic pressure, osmotic pressure, and electric potential gradient changes, which are known to affect their metabolism [4]. Overloading of cartilage causes injury (under 50–70% strain) and cell death (under 70–90% strain) [19]. The optimal biomechanical conditions for in vitro cartilage tissue engineering likely depend on the desired outcome (e.g., homeostasis, maturation, or mineralization) [20]. The cell response to biomechanical stimulation depends on the culture conditions, including the type of scaffold and time of force administration [21].

Two types of biomaterial supports can be used for cartilage regeneration: hydrogels and microporous scaffolds [22]. Hydrogels are polymers with the ability to absorb high water content and a structure similar to that of the native tissue [23]. Hydrogels are biocompatible and can be used as substrates in soft tissue engineering [24, 25]. In addition, the structure can be modified by creating a composite with other polymers or nanoparticles [26, 27]. Various hydrogels, including the biomedical polymers of gelatin-methacrylate(GelMA), hyaluronic acid, collagen, chondroitin sulfate, chitosan, and agarose, have been used in cartilage tissue engineering and regeneration [28–33]. It was also shown that by embedding chondrocytes within the hydrogel, the phenotype and morphology of the cells are maintained in normal conditions [34]. However, the main limitation of hydrogels is their low strength to forces. Therefore, as a biomaterial in a physiological scenario for cartilage repair, the characterization of additional favorable properties is desired, such as bioadhesion to cartilage, the ability to embed high cell densities while minimizing exogenous cell invasion, and the maintenance of its mechano-resilience under simulated joint load [35].

As mentioned earlier, the physical and chemical characteristics of the substrate can affect cell fate, as cells can translate and sense biomechanical cues in their environments [36]. To mimic native tissue in in vitro tissue engineering, it is necessary for the tissue engineering scientists and engineers to understand these signals and copy the stimulation occurring in native tissue. In this article, we review different signaling pathways and applications of biomechanical stimulations in hydrogel-based cartilage tissue engineering, as shown in (Fig. 1B).

### Signals for tissue regeneration

#### Cell Signaling in tissue engineering

Tissue engineering scaffolds are ideal when they are biocompatible and can mimic the native tissue microenvironment and biomechanical properties. In addition, the biodegradability of a scaffold should be considered based on its application to tissue engineering. Stem cell fate can be regulated by interactions between the scaffold microenvironment and cells. These interactions mainly include chemical and physical signals [37]. Therefore, to study the characteristics of a hydrogel scaffold for specific applications, it is necessary to understand the underlying mechanisms of chemical and physical cues affecting cell behavior and other living components [37].

### Biochemical signaling

A variety of biochemical factors can affect cell functions, including the chemical and biochemical properties and morphologies of the ECM material surface, combination of bioactive materials, cell adhesion proteins and peptides, cell coculture, and cell adhesion [38]. For example, stem cells respond to growth factors. Several growth factors, including platelet-derived growth factor (PDGF), insulin-like growth factor(IGF)-1, hepatocyte growth factor(HGF), bone morphological proteins(BMP), transforming growth factor(TGF), fibroblast growth factor(FGF), epidermal growth factor(EGF), and angiopoietin are among these types [39, 40]. Growth factors have also been used to induce cell differentiation and proliferation. Stem cells can differentiate into vascular endothelial cells when certain growth factors such as VEGF are present [41]. As for heart repair, the literature reports that injection of VEGF-gene modified MSC into the heart followed by myocardial infarction increased cell implantation and improved cardiac function compared to unmodified MSC [42]. Regeneration fields, such as bone and cartilage regeneration, also use growth factors. A wide range of cytokines are known to promote bone formation, including BMP, PDGF, TGF-beta, FGF, and IGF; or BMP is known to induce chondrogenic and osteogenic differentiation in mesenchymal stem cell [43–45]. The characteristics of the biomaterial are also important when it comes to cell fate affected by biochemical signaling [46]. For example, cell adhesion is dependent on the biomaterial species and compositions as well as cell adhesion peptides and proteins used as the substrates, which can affect cell differentiation, migration, and proliferation. Peptides such as RGD, LDV, YIGSR have been synthesized and applied for cell adhesion in tissue engineering. Cell membrane receptors, such as integrins, are links between the cell and its microenvironment, and are important for cells to be connected to the ECM. In addition, the biomaterials can cause cell toxicity and lead to apoptosis depending on their species and compositions [47]. However, owing to unknown signaling pathways in cells, it is challenging to guide and manipulate cell fate.

### Biomechanical signaling

Development of advanced fabrication devices using various methods has enabled scientists to overcome some of these challenges [48]. A variety of scaffolds, microchips and bioreactors with different features and functions have been developed via micro/nano engineering methods to study the effect of biomechanical cues in the microenvironment on cell fate in tissue engineering [49–53]. As mentioned above, receptor-ligand interactions, ion channel gates, and other biomechanical cues can be detected by cells as they can convert external stimuli in their environment, such as scaffold properties, substrate topography, and applied tension and compression, into electrochemical responses [54]. These microenvironments can be stimulated and controlled by biomechanical signals from a bioreactor such as compression with static, dynamic and shear stress as well as electrical stimulus. This indicates that externally applied biomechanical cues cause a potential change in the cell membrane and lead to electrochemical activities. Recently, investigating cell responses to biomechanical factors in the environment has become a topic of interest because it is possible to direct cell fate in a specific and desired application [55].

### Stimulation signaling in cartilage tissue engineering

**Fig. 2:**
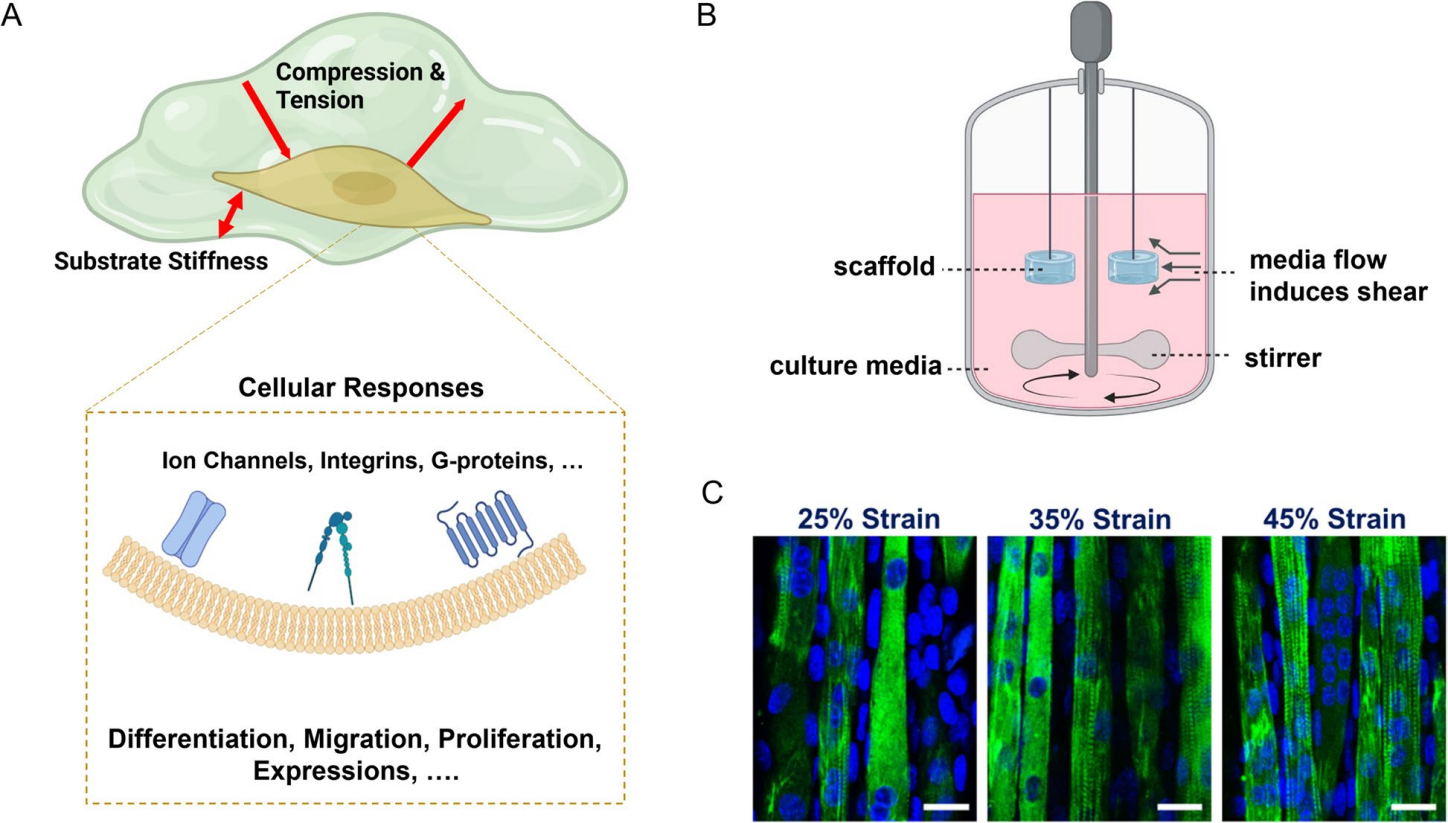
**A** schematic of biomechanical factors affecting encapsulated cell leading to the initiation of biochemical signaling pathways and different cellular responses, **B** mechanism of applying shear stress on hydrogel-based scaffold in a bioreactor, **C** myoblast maturation with a striated pattern under different strain forces observed with a confocal microscope, derived from [65]

In cartilage tissue engineering, biomechanical stimulation is used to develop and maintain the function of cartilage tissue, which is important for tissue transplantation [56–58]. The biomechanical environment under in vivo conditions, such as fluid shear, compression, and hydrostatic pressure, and cyclic load bearing should be considered when developing engineered articular cartilage. Various types of stimulating loads or biomechanical cues on cells, leading to the initiation of biochemical signaling pathways (Fig. 2A). Bioreactors are also designed to apply adequate 3-dimensional biomechanical stimuli like native cartilage for preferable creating engineered in vitro tissues. Petri-dishes are utilized 2-dimensionally to provide nutrition as a basic static bioreactor [59]. However, simulation to the cells of biomechanical forces by bioreactors such as pressure or tension is required for cartilage tissue regeneration by providing mechanisms such as stirring, compression or rolling. The native cartilage stress state is reflected and controlled by each of the applied forced by the bioreactor. By using bioreactors for cartilage tissue construction, it is not only possible to apply noncontact forces, such as magnetic or electric field forces, but also to control environmental conditions, such as hydrogel species and composition, ions, pHs and temperatures. It is important to note that bioreactors in cartilage tissue engineering have two main advantages. A bioreactor can be used to mimic the physical and biomechanical conditions necessary for cartilage growth and development. Alternatively, we can measure the online tissue status and behavior of chondrocytes using digital image processing technology to improve cartilage development in vitro by assessing the impact of different culture conditions [60]. Physiological loading bioreactor systems have been adopted for cultivating engineered cartilage tissues with load-bearing capabilities under the concept of cartilage functional tissue engineering (FTE). Various in vitro and in vivo studies have demonstrated that biomechanical loading maintains articular cartilage [61]. Through dynamic compression loading applied to engineered constructs using agarose as a scaffold material, extracellular matrix composition can be modulated, resulting in cartilage tissues with a Young's modulus close to native values [62]. Bian et el. used agarose hydrogel seeded with adult canine chondrocytes that were subjected to dynamic loading to determine the efficacy of a modified FTE protocol. In their study, the dynamic loading of constructs using bioreactors was applied in unconfined axial compressive deformational loading and sliding contact loading methods for 3 h each day (Fig. 2B) [63]. In another study, Tran et al. have tried to centrifuge a high-density chondrocyte resuspension on an agarose layer in order to create tissue-engineered cartilage from porcine chondrocytes without using a scaffold. They have also increased the biomechanical and biochemical properties of their constructs by culturing them in a bioreactor to apply biomechanical stimulation. With this method, sizeable tissue-engineered cartilage can be produced from porcine chondrocytes [64].

Following section focuses on hydrogels designed for delivering biomechanical signals to cells encapsulated in hydrogels in tissue engineering applications.

### Hydrogels to deliver biomechanical cues

Both natural and synthetic hydrogels can be used as an ECM to deliver biomechanical cues to the cells. Generally, natural polymer-based hydrogels exhibit weaker biomechanical properties with better biocompatibility, whereas synthetic polymer-based hydrogels exhibit higher mechanical stability with better design ability. However, altering the constituent polymer compositions, functional groups, molecular weight, and crosslinking method can change the final biochemical, morphological and biomechanical properties of hydrogels [66].

### 3D culture microenvironment in cell-encapsulated hydrogels

Owing to the differences between in vitro 2D and 3D cell environments, engineering of 3D scaffolds has emerged as a novel approach in tissue engineering. Among all the biomaterials used in this approach, hydrogel-based scaffolds have shown great potential for easy cell encapsulation with biocompatibility and biomimetic of ECM properties, as they can mimic the 3D cell environment of natural tissues. The hydrogel-based scaffold should induce changes in cell functions to regulate cell fate (proliferation, migration, and differentiation) by withstanding biomechanical loads and allowing nutrient transport [67, 68]. To design a hydrogel scaffold that delivers biomechanical stimulation to cells, it is important to study its polymer species and their properties and stimulation approaches. The results of 2D studies of different cells, such as mesenchymal stem cells (MSCs), showed that cell behavior differs by manipulating the mechanical properties of scaffolds, such as stiffness and mechanical loads [69, 70]. In 3D models, the development of hydrogel-based scaffolds allows researchers to tune the biocompatibility, water content, biomechanical loads, and other mechanical and physical properties to mimic a 3D cell environment of tissues.

The most important method for manipulating the degrees of stiffness is altering the polymer species, composition, concentration of the hydrogel. Glioblastoma is an aggressive type of cancer that is resistant to conventional treatments. To study the most invasive phenotype of glioblastoma, Erickson et al. fabricated chitosan–hyaluronic acid polyelectrolyte scaffolds with various stiffness values. The results indicated that glioblastoma cells were more resistant to chemotherapy when cultured in scaffolds with higher stiffness [71]. Pore size and shapes are other factors that affect the cell fate. Brennan et al. observed that human bone marrow stem cell scaffolds showed better results with a 100 μm pore size poly(ε-caprolactone) scaffold than 200 and 300 μm, leading to collagen and mineral deposition of hMSCs into the scaffolds. The pore size decrease caused the scaffold stiffer, which had a positive effect on the osteogenic differentiation of the stem cells, leading to deposition of collagen and mineral into the scaffolds [72]. The use of different cross-linking agents (physical and chemical) for hydrogels also leads to different stiffness values. Sridharan et al. used three different crosslinkers for collagen to study macrophages behavior on scaffolds. As shown in the results, macrophage responses to chemical and physical crosslinkers are not the same, and they can be chosen based on the application. They reported that EDAC (1-Ethyl-3-(3-dimethylaminopropyl)carbodiimide) cross-linking promotes both proinflammatory and anti-inflammatory responses from macrophages. That makes it a suitable cross-linking agent for tissue engineering applications, but genipin cross-linked scaffolds may be suitable for applications where any inflammatory response needs to be suppressed [73]. Hydrogel properties such as polymer species, composition, molecular weight, crosslinking density, mechanical heterogeneity and related environments such as time, pH, temperature, and addition of nanomaterials such as calcium phosphate, carbon nanotubes and fibers are among other methods used to alter the stiffness of hydrogel-based scaffolds [74].

Biomechanical loads are other parameters that cells encapsulated in hydrogels can sense, and it is important to study their responses as ligaments, tendons, cartilage, heart beating, muscles, and many other tissues exposed to these mechanical loads. Kong et al. developed a microdevice that mimics the biochemical microenvironment of cardiac tissue. This device applies compression to gelatin-methacrylate(GelMA) hydrogel scaffolds containing cardiac fibroblasts. As revealed by their study, cardiac fibroblasts can spread into the hydrogel-based scaffold and proliferate under cyclic compression. Additionally, a transition of phenotypes from fibroblast to myofibroblast was observed under 15 – 20% of strains [75]. In another study by Chen et al., myoblast cell encapsulated GelMA microfibers were treated with different ratios of uniaxial stretching. Myofiber growth and contractility increased depending to the stretching ratio (Fig. 2C) [65]. The effect of cyclic tensile loads on hMSCs cultured in poly(ethylene glycol)-based hydrogel scaffolds was also examined. No difference was observed in the cell populations in both the control and mechanically stimulated samples. However, cells under cyclic strains tend to express tendon/ligament fibroblastic genes [76]. In another study by Jeon et al., hMSCs were encapsulated in an alginate-gelatin hydrogel to study the impact of biomechanical cues on cell fate. Cyclic loadings in this research led to osteogenic differentiation and enhanced proliferation [77]. Rinoldi et al. observed enhanced cell fate as the result of biomechanical and biochemical stimulation on a cell-laden hydrogel thin layer on fiber substrates [78]. In another study, Lin et al. synthesized methacrylated hyaluronic acid to encapsulate MSCs, and the cell-containing scaffolds were exposed to compression loading. 7 days preconditioning incubation of MSCs in the chondrogenic induction medium containing 10 ng/mL TGF-β1 and another 7 days growth in basal growth medium (α-MEM) were administered before hydrogel encapsulation. Synergistic effects on chondrogenic differentiation and more viability were observed as a consequence of precondition and mechanical loads [79]. Overall, applying mechanical stretches and compressions, shear stress, and stress/strains to hydrogel-based scaffolds, as well as altering their stiffness, are methods for manipulating cell fate in tissue engineering applications [80].

As mentioned earlier, 3D-culture of cells in a hydrogel scaffold, subjecting it to suitable biomechanical stimuli before implantation, is one approach to cartilage tissue regeneration. Another approach is using injectable hydrogels to make a 3D microenvironment in the human body. It is an emerging strategy that can deliver growth factors and therapeutic cells to the defect area, also it is less invasive and can easily fill the irregular shape and depth of the defect area [81, 82]. Several natural polymers have been utilized in injectable systems, including collagen, chitosan, gelatin, alginate, hyaluronan, elastin, heparin and chondroitin sulfate as examples [83–91]. A wide variety of injectable systems have been studied, but fibrin-, hyaluronic-, gelatin- and alginate-based hydrogels have received the most attention. There are several methods for in situ gelation, including photo, chemical and enzymatic crosslinking, pH-induced and temperature-induced gelation, ionic and hydrophobic interactions [83]. Table 1 shows some examples of cell-encapsulating hydrogels used in cartilage tissue engineering.

**Table 1:** Examples of cell-encapsulating hydrogel used in cartilage tissue regeneration. Further detailed information of stimulation, cells and gels is described in Table2

Hydrogel	x-linking method		Stimulation	Cell encapsulation	Ref
	Chemical	Physical			
HA-PEG4-DBCO	O		N/A	in situ	[92]
HA-GM	O		N/A	*syringe*	[93]
Chondroitin sulfate/pullulan		O	N/A	in situ	[94]
PEG/Dextran/ Fibrin		O	Different stiffness	in situ	[95]
HA/gelatin	O		Electrical	in situ	[96]
Collagen/Chondroitin sulfate	O	O	N/A	in situ	[97]
agarose	O		Mechanical compression	in situ	[98]
Chitosan/HA		O	N/A	in situ	[99]
alginate			N/A	*syringe*	[100]

*N/A* Not Applicable

### Factors affecting cellular responses on biomechanical stimuli-induced cartilage tissue regeneration via cell-encapsulated hydrogels

As articular cartilage is subjected to different biomechanical forces, several in vivo and in vitro studies have been conducted to evaluate the effect of biomechanical stimuli on chondrogenesis. Biomechanical stimulus is a promising tool for designing a biomimetic 3D environment for chondrogenic models [101]. In addition to imitating the natural tissue environment, biomechanical stimuli can 1) help cells penetrate the hydrogel scaffold [102], 2) enhance the mechanical properties of regenerative cartilage tissues [103, 104], and 3) enhance nutrient delivery and local oxygen availability for cells in hydrogels [105].

It is well recognized that articular cartilage has different zones as described in previous, and the properties of each zone differ [106, 107]. The in vivo biomechanical load condition in the cartilage varies in the zones, with the strain in the superficial zone being the highest and that in the deep zone being the lowest. Chondrocytes on the surface endure both compressive and shear strains, but stimulation in the deeper layers is mostly compressive. This biomechanical load causes a different elastic modulus; in the superficial zone, it is less than that in the deep zone [108, 109]. Several factors should be considered to imitate the natural properties of the cartilage environment and determine the ideal setting for biomechanical stimuli to promote cartilage growth [110]. In following section, the factors affecting cellular response is discussed for some significant recent studies.

### Cell type

For 3D-cell-laden hydrogels, several cell types, such as different types of stem cells or specific tissue cell types, or their co-cultures have been used for tissue regeneration. Stem cell source is a crucial factor that should be considered when designing cartilage-engineered constructs. MSCs are well-known for being a great cell source owing to easiness of cell isolation, ability to self-renew, and chondrogenic differentiation. Comparing all MSC source, synovium-derived mesenchymal stem cells (SMSCs) are one of the promising stem cells for cartilage regeneration [98, 111]. It should be also considered that during chondrogenic development, BMSCs exhibit a much higher tendency for osteogenesis [112]. Ossification and hypertrophic differentiation during chondrogenic induction are yet another significant issue in long-term MSC cultures [113]. Researchers have assessed several cell types for their chondrogenic models because each type of stem cell responds differently to biomechanical stimulation. In the study by Luo et al., two different sources of stem cells were utilized to study the effect of dynamic culture on cells encapsulated in agarose hydrogel: Porcine BM derived stem cells (BMSCs) and fat pad(FP)-derived stem cells (FPSCs); BMSCs were a better choice to attain cartilage matrix with better biomechanical properties, suppressed hypertrophy, and increased matrix accumulation compared to FPSCs [114]. Another study by Carroll et al. showed that adding physiological amounts of hydrostatic pressure (HP) to both infrapatellar fat pad-derived multipotent stromal cells (FPSCs) and multipotent stromal cells derived from porcine bone marrow (BMSCs) encapsulated in agarose hydrogels enhanced their biomechanical function and promoted the growth of a more stable cartilaginous phenotype [115]. In both groups, HP enhanced sulfated glycosaminoglycans (sGAG), but in FPSCs, HP caused less collagen accumulation compared to BMSCs [115]. HP can maintain the cartilaginous phenotype in FPSCs in the absence of chondrogenic medium (containing transforming growth factor, TGF-β3) compared with free swelling samples [115]. Terminal differentiation, a hypertrophic phenotype and precursor of endochondral ossification, is the greatest obstacle to the use of MSCs. In this regard, a study evaluated the compression-loading effect on the inhibition of hypertrophy in MSC encapsulated in agarose hydrogels. They described a biomimetic hydrogel with PEG as the main component, and two extracellular matrix analogs (cellular adhesion peptide based on RGD and a sGAG based on chondroitin sulfate) were integrated into the PEG hydrogel. This hydrogel enhanced chondrogenesis by upregulating collagen type II, but due to the expression of collagen X, the hypertrophy phenotype of cells was evident. However, applying biomechanical stimuli in certain regimes, (10% 0.3 Hz) and (5% 1 Hz), resulted in a more stable chondrogenic phenotype by inhibiting collagen X expression in the constructs [116].

### Pre-culture conditioning before biomechanical stimulation

Throughout the chondrogenic differentiation process, stem cells' mechanosensitive response may vary [117]. Therefore, the duration of biomechanical stimuli and preculture before applying biomechanical stimuli is a crucial factor for cell fate. Dynamic compression at an early stage with limited preculture time downregulates chondrogenic markers such as COL2 and SOX9 [98]. Loading without preculture downregulates the sulphated glycosaminoglycans amount [118]. In the research conducted by McDermott et al., prior to two weeks of either static culture or dynamic compression, human bone marrow stromal cells (hMSCs) were cultured in fibrin hydrogels under chondrogenic priming conditions different times including 0, 2, 4, or 6 weeks. Their findings showed that a low priming time preserves chondrocyte homeostasis, and a long priming time causes cartilage maturation [20].

### Cell density

Cell density is another crucial factor that regulates the effect of biomechanical stimuli on chondrogenesis, because cell density directly affects the density of progenitor cell condensation in cartilage formation. Bian et al. observed that the lower cell density encapsulated at the initiation time caused a delay in the effect of mechanical load on the construct compared to the high cell density in the long-time culture (70 days). The expression of hypertrophic markers by human MSC is considerably reduced by dynamic compressive loading, and the degree of calcification in MSC-seeded HA hydrogels is suppressed [113]. Therefore, when using MSCs, it is essential to have a proper cell-seeding density for cartilage tissue creation.

### Growth factors

Biomechanical load can be used individually or in combination with other factors such as chondrogenic growth factors. TGF-β family; such as TGF-β1 and TGF-β3; is one the most popular growth factor for inducing chondrogenesis [119, 120]. Biomechanical stimuli are a promising tool for inducing chondrogenesis even in the absence of TGF-β [121]. Huang et al. showed that although TGF-β1 alone could be a promising tool to enhance chondrogenesis, collagen type II gene expression in rabbit BM-MSCs was more efficiently induced by the combination of cyclic compressive loading and TGF-β1 therapy, rather than by TGF-β1 alone. The effects of biomechanical stimulation on different stem cell types vary. Bone marrow MSC encapsulated in hydrogels express chondrogenic markers under biomechanical stimulation in the absence of TGF-β1. However, the expression of chondrogenic genes was downregulated in hEBd (human embryoid body-derived) stem cells in the absence of TGF-1. However, biomechanical compression promoted chondrogenic differentiation of hEBd cells after two weeks of TGF-1 conditioning. In particular, stimulation of 2.0–2.5 h (with 10% strain and 1 Hz) seems to be ideal for both cell groups in terms of their chondrogenic development, as demonstrated by the increase in gene expression and/or ECM formation [104]. Ge et al. investigated the effect of biomechanical dynamic compression on encapsulated human synovium-derived mesenchymal stem cells in agarose hydrogels, with and without TGF- β3. Pro-chondrogenic genes were upregulated by biomechanical compression alone, but the TGF- β3 therapy caused these genes to be upregulated much more. Regardless of TGF- β3 presence or absence, mechanical compression had an anti-hypertrophy impact [98].

In another study, Antunes et al. evaluated the effect of biomechanical and FGF-18v biochemical signals on primary bovine articular chondrocytes embedded in a fibrin-hyaluronan hydrogel. The moderate multiaxial load applied to the constructs showed an increase in sGAG/DNA. A significant effect of FGF-18v was observed only during loading, but not at rest. It increased cartilage ECM components such as ACAN, COMP, COL2, and PRG4, and decreased joint destruction factors, including MMP-9 and MMP-13 [122].

Aisenbrey et al. used a photo-clickable hydrogel for encapsulating induced pluripotent mesenchymal progenitor cells (iPS-MPs), and the individual and synergistic effect of TGF-β3, BMP2 and dynamic compression were evaluated. The best condition to induce stable chondrogenesis was the combination of TGF-β3 and compression loading, which also led to the inhibition of hypertrophy. Positive TGF-βRI expression with load enhanced Smad2/3 signaling and low SMAD1/5/8 signaling was observed. In summary, this study reports a promising cartilage-mimetic hydrogel for iPS-MPs that when combined with appropriate biochemical and biomechanical cues induces a stable chondrogenic phenotype [123].

### Biomechanical stimulation (Load magnitude and frequency)

The scaffold could be subjected to a wide range of frequencies and loads. This range should be selected based on the target tissues. It has been proven that, for cartilage tissue, the load and frequency of loading should resemble human walking. Based on research conducted by Natenstedt et al., the best condition for cartilage tissue is 5–10 MPa with a frequency of 1 Hz for a week or more [124]. Kowsari-Esfahan et al. used microfluidic device for unidirectional compressive stimulation for cells and showed that 10% strain (among 0, 5, 10, 15, and 20%) was the optimal to induce chondrogenesis in encapsulated ADSCs in alginate hydrogel [125].

### Type and number of applied biomechanical forces

Different bioreactors have been utilized to study the effects of external biomechanical stimuli on proliferation and chondrogenesis, including shear bioreactors, compression and perfusion, and hydrostatic pressure (HP) bioreactors [126]. Some studies have applied one biomechanical stimulus, and some have used hybrid bioreactors to apply multiple loads to the designed construct [127, 128]. In a study by Ogura et al., human articular chondrocytes encapsulated in agarose hydrogel and hydrostatic pressure and/or deviatoric stress were administered singly or in combination. They showed that each of these biomechanical stimulations played an independent role in altering cell proliferation and metabolic functions. In a cell construct, HP will be helpful promotes the synthesis of cartilage ECMs, whereas deviatoric stress has an ECM-catabolic effect [129].

Cochis et al. tested a novel thermos-reversible methyl-cellulose (MC) hydrogel. MSC were encapsulated in the MC solution and added to the polyurethane (PU) porous scaffold. The combination of shear and compression leads to a significant increase in chondrogenic gene expression and increased collagen 2 and GAGs [127]. In another study, a perfusion/pressurized bioreactor that combines shear stress and oscillating hydrostatic pressure (OHP) was used to study the effect of shear stress and shear stress/OHP with and without CM supplementation for cartilage regeneration in different culture techniques, including micro-mass, pellet and encapsulation. Bovine articular chondrocytes encapsulated in 2% agarose hydrogel were used. Regarding biochemical and biomechanical parameters, samples cultivated in pellets and micro-mass do not differ noticeably. However, agarose encapsulation significantly increased GAG and collagen secretion. Shear stress/OHP caused a higher increase in GAG and collagen secretion compared to individual shear stress. Moreover, it suppressed the expression of non-chondrogenic markers, including Collagen X, Collagen 1, and β1 integrin. It also increased the elastic moduli of the samples. CM had no significant effect on chondrogenesis [130]. Shadi et al. produced a chondrocyte-laden decellularized scaffold subjected to dynamic compression and shear stimuli in an ad hoc bioreactor. Decellularized ECM scaffolds appear to provide an appropriate microenvironment for chondrocyte activity. It was also shown that the bioreactor could mimic a load-bearing meniscus during joint movement [21].

Some studies of the biomechanical stimulation of cell-encapsulating hydrogels and cell responses are listed in Table 2 with the information of preculture time, biomechanical stimulation type and frequency, magnitude and its duration as well as cell types employed.

**Table 2:** Mechanical stimulation on cell containing hydrogel and responses

Cell type /Hydrogel	Preculture time	Mechanical stimuli	Duration	Response	Ref
Rabbit BM-MSCs / Agarose	24 h	Compression/10% strain /1 Hz	4 h/day, 14 days	-Aggrecan and collagen type II expression increased in all groups (TGF-β, TGF-β + loading, Loading)—TGF-β1 gene expression in both loading and TGF-β treatment. -No change in DNA content after stimulation.—No collagen type X expression	[131]
Human chondrocytes/ collagen type I	-	Compression/10% strain /0.3 Hz	Continuous, 28 days	-Upregulation of Col II and Col I and MMP-13, down regulation of aggrecan—No change in the ratio of Col II/ Col I mRNA expression. -Enhanced stiffness of construct in loaded sample	[103]
Human synovium-derived mesenchymal stem cells (SMSCs)/ Agarose	1 day and 21 days	10 kPa/ 0.25 Hz	1 h/day till day 28	-Loading after 21 days preculture increased aggrecan and COL2α1 expression level and suppressed hypertrophy markers including COL1α1, COL10α1, MMP13, RUNX2, and ALP	[98]
Chondrocyte/ Fibrin-polyurethane	5 days	10–20% strain with 0.5 Hz and simultaneous shear motion by ball oscillation at ± 25° and 0.5 Hz	1 h loading, twice per day (8 h free swelling between loading cycles) over 5 consecutive days	-Upregulation of COMP and PRG4 gene -Increased Col2/Col1 & ACAN/VCAN, MMP3 and MMP13 remained relatively stable. -No effect on GAG production per cell	[132]
iPS-MP/ photo crosslinkable hydrogel (8-arm PEG-norbornene, ChS-SH, CRGDS (Genscript), PEG dithiol, and Irgacure 2959)	7 days	Intermittent unconfined dynamic compressive strains applied at 5% peak to peak strain (2.5% amplitude strain) at 1 Hz with 23 h of rest under a tare strain of < 0.1%	1 h/day for 3 weeks	-Synergistic effect of dynamic loading and TGF-B3 was the best condition to induce stable chondrogenesis and inhibit hypertrophy	[123]
Human bone marrow stromal cells (hMSCs) /Fibrin hydrogel	0, 2, 4, or 6 weeks	1 Hz to an amplitude of 10% strain, after a 0.01 N preload	2 h/day, 5 days per week/2 weeks	-Enhanced COL2A1 and ACAN mRNA expression in more than 2 weeks priming samples -Increased COL2A1 and COL10A1 and suppressed SOX9 expression with no change on ACAN after immediate mechanical loading (No chondrogenic priming) -Enhanced GAG and collagen deposition and mechanical properties by increasing priming time	[20]
Primed MSCs/MeHA hydrogel (In vivo)	1 day	10% peak compressive sinusoidal strain at 1 Hz superimposed on a 5% compressive tare strain	4 h/day and 5 days per week/14 days	-Dynamic loaded constructs showed cartilage defect healing in Rat and nude mice	[79]
Human articular chondrocytes (in vivo)/Fibrin	-	Perfusion, 2.5 µL/s	21 days	-Accumulated and more distributed GAGs in the perfused samples -Accumulation of collagen type II in perfused gels	[133]
Human articular chondrocytes /Agarose	-	Hydrostatic pressure and/or deviatoric stress	4 days loading and 3 days static culture (7 days in total)	-Cell proliferation with mechanical loading -Increase in aggrecan and collagen type II at day 7 in HP sample -Increase in aggrecan and collagen type II at day 4 in deviatoric stress and combined HP/ deviatoric stress sample	[129]
Primary bovine articular chondrocytes Fibrin-hyaluronan based hydrogel	5 days	10%/0.5 Hz compression, 25°/0.5 Hz rotation	1 h/day, 14 days	-sGAG/DNA upregulation in loaded samples both in the presence or absence of FGF-18v	[122]
Porcine bone marrow (BM) and infrapatellar fat pad (FP) derived stem cells/Agarose	21 days	Compression 10% strain,1 Hz	1 h/day, 21 days	-Increase in sGAG and collagen content after loading in both cells -An increase in DNA content was in FP samples after loading-Relatively constant DNA content in BM samples after loading	[114]
Porcine bone marrow derived multipotent stromal cells (BMSCs) and infrapatellar FP-derived multipotent stromal cells (FPSCs)/ Agarose	24 h	HP/10 MPa /1 Hz	4h/day, 5days/week for5 weeks	-Increase sGAG in both groups -Enhanced collagen accumulation in BMSCs -HP reduced hypertrophic differentiation and endochondral ossification in BMSC -HP caused a stable cartiligous phenotype in FPSC group	[115]
MC-PU/MSC	2 days	Compression and shear strain (Shear ± 25° at 1 Hz/Compression 10–20%, 1 Hz)	1 h/day for 21 consecutive days	High collagen II expression and low COL-X expression in loaded sample	[127]

### Other studies

**Fig. 3:**
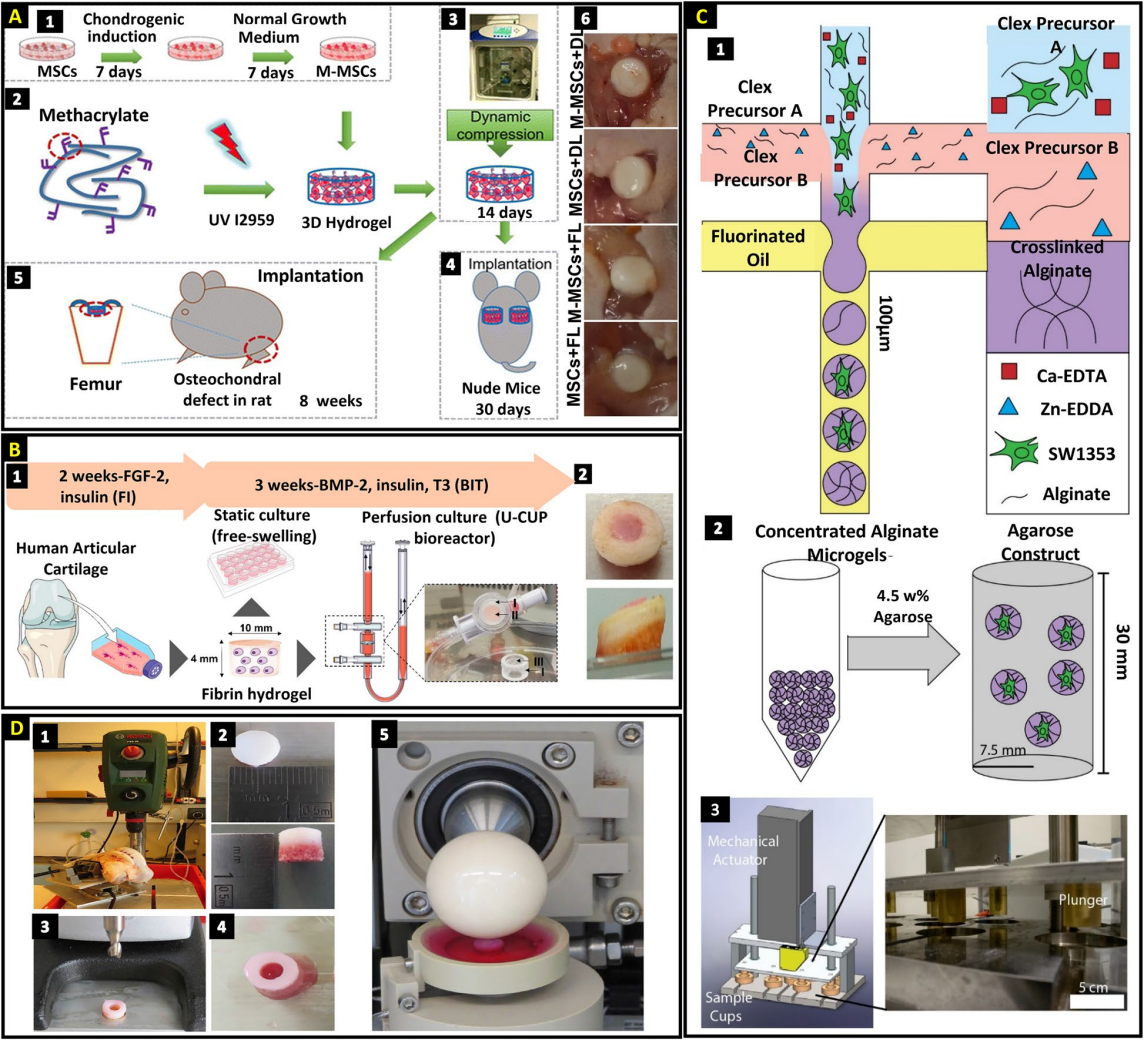
**A** 1: Procedure for chondrogenic preconditioning to obtain manipulated mesenchymal stem cells (M-MSCs), 2: Methacrylated hyaluronic acid (MeHA) loaded with cells undergoes UV-initiated x-linking, 3: compressive stress in a bioreactor for the MeHA hydrogel containing cells, 4: The constructions were subcutaneously implanted in nude mice for 30 days following 14 days of culture in a bioreactor, 5: Implantation in osteochondral defect in rats for 8 weeks after culturing in bioreactor for 14 days, 6: Following implantation in naked mice, a representative gross view of the resulting constructions is shown, reproduced content [79]. **B** The setup of the in vitro experiment and perfusion chamber, 1: Extraction and amplification of chondrocyte followed by seeding cells in fibrin hydrogel for 3 weeks in both static (well plate) and perfusion bioreactor while being exposed to BMP-2, insulin, and T3 (BIT), 2: Top and side views of macroscopic images displaying the cartilage gel centered in the human osteochondral block, reproduced content [133]. **C** 1: Schematic of the production process of alginate microgels, 2: Mixing concentrated alginate microgels with agarose hydrogel after 9 days culture, 3: Appling cyclic strain (5 ± 2%) to the agarose constructs containing alginate microgels in the custom-made bioreactor, reproduced content [17]. **D** 1–4: Osteochondral harvesting and defect creations, 5: osteochondral defect models are stimulated using a joint bioreactor with joint-specific biomechanical stimuli, reproduced content [132]. Reproduced content is open access-Attribution 4.0 International (CC BY 4.0)

In an in vivo study by Lin et al., a scaffold composed of methacrylate hyaluronic acid hydrogel was used to encapsulate bone marrow-derived MSCs (Fig. 3A). In the first step, manipulated mesenchymal stem cells (M-MSCs) were obtained, and the cells were loaded in methacrylated hyaluronic acid. Afterwards, the constructs were dynamically loaded in chondrogenic media using the CartiGen Bioreactor System for 14 days. The constructs were subcutaneously implanted in nude mice for 30 days and osteochondral defects in rats for 8 weeks. Thirty days after implantation in nude mice, dynamical loading promoted neocartilage production in the hydrogel encapsulated with M-MSCs (Fig. 3A (6)). A rat model of osteochondral defects showed improved cartilage healing as well after 14 days implantation [79].

In an in vivo study by Dufour et al., synergistic effect of three combined soluble factors (BMP-2, insulin, and tri-iodothyronine, that is, BIT) and perfusion bioreactor on human chondrocyte cell encapsulated in the fibrin hydrogel were evaluated. Firstly, the cells were amplified in chondrogenic medium, followed by seeding cells in fibrin hydrogel for 3 weeks in both static and perfusion bioreactor while being exposed to BIT (Fig. 3B (1)). Treatment with a perfusion bioreactor before implantation caused integration with the native tissue (Fig. 3B (2)), as well as the secretion of cartilage matrix components such as type II and type VI collagen [133].

In a recent work and strategy used by Fredrikson et al., single chondrocytes were encapsulated in alginate microgels to mimic the PCM and microenvironment of a single chondrocyte cell using drop-based microfluidics (Fig. 3C (1)). Afterwards, single-cell microgels were embedded in agarose hydrogel to make the whole construct more accurate for the in vitro chondrocyte mechano-transduction model (Fig. 3C (2)). Higher collagen VI formation is reported in chondrocyte embedded microgels. The microgels displayed distinct metabolomic profiles from the uncompressed and monolayer controls after dynamic compression (Fig. 3C (3)) [17].

Behrendt et al. encapsulated human bone marrow–derived mesenchymal stem cells in tyramine-modified hyaluronic acid (HA-Tyr) hydrogels for osteochondral defects repair strategies. This bio-adhesive material is a promising carrier under biomechanical conditions for activating endogenous TGF-β1 in cells. For multiaxial loading, cell-laden hydrogels were subjected to 10% compression superimposed onto a 0.5-N preload and shear loading (± 25º) at 1 Hz for 1 h per day, 5 times in a week for 28 days [35].

As an ex vivo culture model, an osteochondral defect model was developed in a bioreactor that replicates the multi-axial motion of an articulating joint [132]. Osteochondral defects were created in the explant, filled with chondrocyte-laden fibrin-polyurethane scaffold, and subjected to confined shear and compression loads (Fig. 3D). Owing to the confined model, a hydrostatic pressure can also be built. More cartilage matrix deposition and chondrogenic differentiation occurred in the loaded samples than in the unloaded samples.

### Summary and future direction

Owing to the importance of cartilage tissue regeneration, several studies have been performed to design models to imitate the natural cartilage environment and to match the native whole joint situation; however, none of them could completely mimic the entire complex structure of articular cartilage.

It is well recognized that biomechanical stimuli are a prominent tool for controlling the homeostasis and maturation process of chondrocytes. Along with biomechanical stimulation, several other factors should be considered including cell type and source, cell density, scaffold composition and properties, biochemical signals like growth factors, preculturing time before applying biomechanical stimuli, biomechanical stimulus type, bioreactor design, stimulation period, frequency, and loading patterns and frequency. Many studies have endeavored to determine optimized loading parameters for chondrogenic differentiation, especially in terms of magnitude and frequency. More studies should be conducted on other parameters, including loading initiation time and loading duration. A popular in vitro stimulation for cell-seeded biomaterials is cyclic compression loading because biomechanical stimulation within the joints is closest to this mode. However, the use of a hybrid bioreactor and synergistic effect of multiple and diverse loadings on the construct should be considered.

Since chondrocyte phenotypic expression can be preserved in agarose culture and its biochemical and biomechanical properties are analogous to those of natural cartilage, agarose hydrogel is mostly used in biomechanical loading studies. Therefore, as a construct for cartilage regeneration under biomechanical stimuli, more complex and advanced biomaterials mimicking native tissue should be considered. Moreover, few studies have attempted to replicate implantable cartilage constructs for clinical use.

Totally the advancement of cell-based therapy for repairing cartilage tissue can be achievable by chondrogenic preconditioning, implantable scaffold together with biomechanical stimulation to be able to load bearing in physiological condition.

## Data Availability

Not applicable.

## Abbreviations

BMP: Bone morphological proteins
ECM: Extracellular matrix
EGF: Epidermal growth factor
GelMA: Gelatin-methacrylate
FGF: Fibroblast growth factor
HA-Tyr: Tyramine-modified hyaluronic acid
HGF: Hepatocyte growth factor
HP: Hydrostatic pressure
IGF: Insulin-like growth factor
MC: Methyl-cellulose
MeHA: Methacrylated hyaluronic acid
M-MSCs: Manipulated mesenchymal stem cells
MSC: Mesenchymal stem cells
OHP: Oscillating hydrostatic pressure
PCM: Pericellular matrix
PDGF: Platelet-derived growth factor
PU: Polyurethane
sGAG: Sulfated glycosaminoglycans
TGF: Transforming growth factor
