# Correction: Progress in biomechanical stimuli on the cell-encapsulated hydrogels for cartilage tissue regeneration

**DOI:** 10.1186/s40824-023-00414-6

**Published:** 2023-07-24

**Authors:** Shiva Taheri, Hanieh Sadat Ghazali, Zahra Sadat Ghazali, Amitava Bhattacharyya, Insup Noh

**Affiliations:** 1 Convergence Institute of Biomedical Engineering and Biomaterials, Seoul National University of Science and Technology, Seoul, 01811 Republic of Korea; 2 Department of Nanotechnology, School of Advanced Technologies, Iran University of Science and Technology, Tehran, 1684613114 Iran; 3 Department of Biomedical Engineering, Amirkabir University of Technology, Tehran, 158754413 Iran; 4 Functional, Innovative, and Smart Textiles, PSG Institute of Advanced Studies, Coimbatore, 641004 India; 5 Department of Chemical and Biomolecular Engineering, Seoul National University of Science and Technology, Seoul, 01811 Republic of Korea

**Correction: Biomaterials Research 27, 22 (2023)**

**https://doi.org/10.1186/s40824-023-00358-x**

In the article “Progress in biomechanical stimuli on the cell-encapsulated hydrogels for cartilage tissue regeneration”, which was published in Biomaterials Research on March 20th, 2023 [1], we would like to issue the following erratum regarding the publication history.

The article was initially made available as a preprint publication on February 25th, 2023, before its final publication in Biomaterials Research. The content and findings of the article remain unchanged.

We regret any inconvenience or misunderstanding this error may have caused. Please note that this correction is being made to ensure accurate documentation of the publication history and does not affect the integrity or validity of the research presented.
